# A Rare Case of Colo-Colonic Intussusception Caused by Colonic Submucosal Lipoma

**DOI:** 10.7759/cureus.23600

**Published:** 2022-03-29

**Authors:** Vishal Busa, Sai Samyuktha Bandaru, Rameela Mahat, Chaitra Janga

**Affiliations:** 1 Internal Medicine, Baton Rouge General Medical Center, Baton Rouge, USA; 2 Internal Medicine, Abington Hospital-Jefferson Health, Abington, USA

**Keywords:** bowel resection, intussusception, lower abdominal pain, colonic intussusception, colonic lipoma

## Abstract

Unlike in infancy, where intussusception is an abdominal emergency, diagnosis of intussusception could be tricky in adults as most of these patients present sub-acutely with vague abdominal symptoms. Early diagnosis could impact these patients significantly in decreasing morbidity and mortality along with reduction in healthcare expenses. Colo-colonic intussusception is rare and accounts for less than 20% of adult intussusception. More than 50% of adult intussusception is caused by mass-like lesions. In such cases, this could be an early presentation of malignant intestinal lesions. Abdominal CT is mandatory in all adult patients; when combined with ultrasound, it has 95.5% accuracy of pre-operative diagnosis. Here we report a case of a 42-year-old female who presented with a two-month history of intermittent abdominal pain; a CT abdomen revealed colo-colonic intussusception which was initially missed on prior imaging. We discuss the importance of considering intussusception as a rare differential of abdominal pain, the need for early diagnosis, and the role of colonoscopy and non-surgical management in adults.

## Introduction

Abdominal pain is the common presentation of intussusception, however, given the rarity of it in adults, the possibility of missing the finding in abdominal imaging leads to misdiagnosis which ultimately attributes these symptoms to a different condition. It is shown that in 22% of cases, an initial abdominal CT could miss intussusception [1]. Therefore, accurate diagnosis needs specific attention to the patient's history and clinical presentation which varies widely from chronic intermittent abdominal pain to an acute small bowel obstruction. Intestinal obstructions are rare and account for < 1% of these cases [2]. Usually, patients present at the physician's office with chronic intermittent pain from weeks to months and persistent symptoms despite using different treatments. In this report, we discuss a case of colo-colonic intussusception with large transverse colon mucosal lipoma as a lead point; we also emphasize the need for keeping it as a rare differential for abdominal pain.

## Case presentation

A 42-year-old female with a past medical history of uterine fibroids presented to our emergency department with complaints of persistent diffuse lower abdominal pain that started more than a month prior to the presentation. The patient’s pain was intermittent in nature with varying intensity associated with mild nausea. She denied any vomiting; there was no association of abdominal pain with food intake, no aggravating or relieving factors. She reported normal bowel movements and flatus. On examination, there was no tenderness noticed on any quadrants. Initial laboratory work-up was non-significant with a normal white cell count. The patient was following her primary care physician one month prior to her presentation due to her persistent abdominal pain; the CT abdomen performed in the outpatient setting was unremarkable. Given her vague and non-specific symptoms, abdominal pain was thought to be related to her fibroids and the patient subsequently underwent a laparoscopic hysterectomy. However, her symptoms still persisted post-surgery which prompted her to visit the hospital.

**Figure 1:**
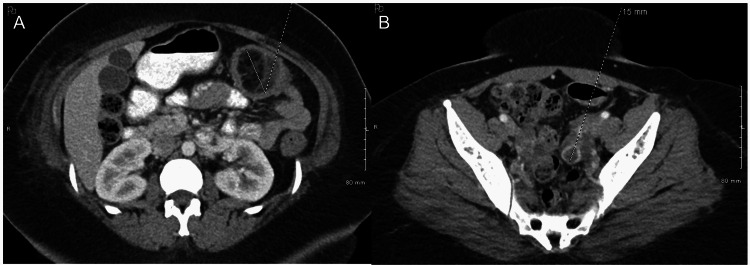
CT image revealed colo-colonic intussusception with lipomatous mass lesion (A, B); dotted lines in figure A are consistent with the target sign

**Figure 2:**
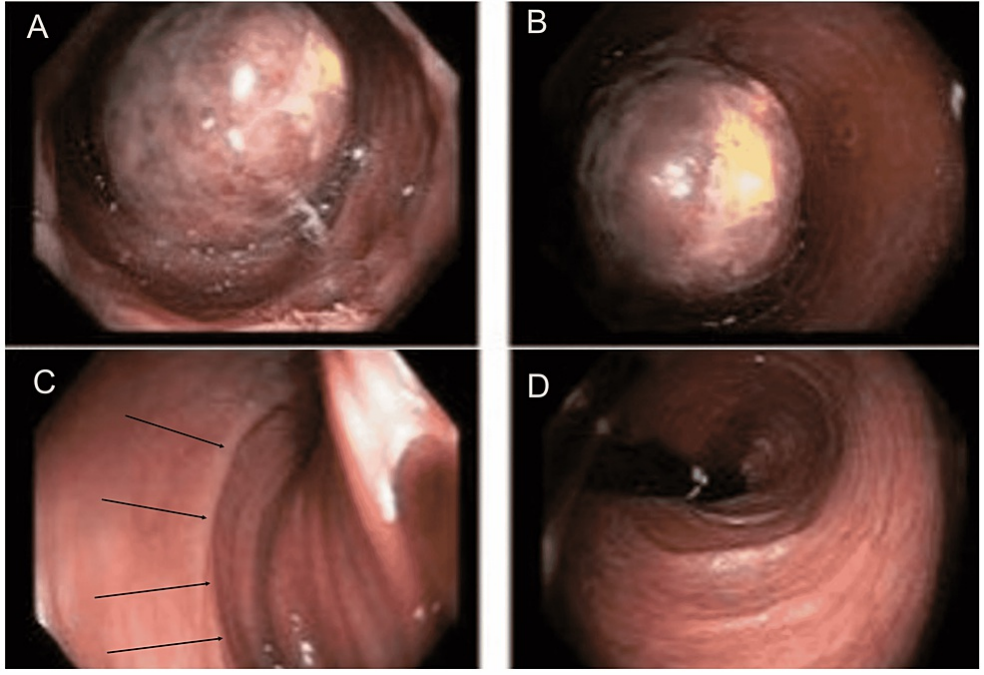
Colonoscopy revealed a large non-bleeding 7-cm mucosal mass of benign appearance found in the transverse colon (A, B); solid black lines in Figure C revealed telescoped segments of the colon; Figure D showed normal colonic mucosa on retroflexion during colonoscopy

CT abdomen revealed a colo-colonic intussusception with a 4.6 cm transverse colon lipoma as a lead point and a moderate inflammation of the bowel involved with the intussusception; no definite pneumatosis or free air was seen (Figure 1). Subsequently, bowel preparation was done and the patient underwent a colonoscopy that revealed a mucosal nonbleeding, non-pedunculated 7-cm mass of benign appearance which was found in the mid transverse colon with partial obstruction (Figure 2). Biopsy was done due to concerns for malignancy as the mass was large and colonic in origin. During the biopsy, the mass appeared to be friable so biopsies were obtained as superficial as possible to minimize the bleeding.

Pre-operative biopsy revealed fibrinopurulent findings suggestive of a lipoma and negative for malignancy. Colonic intussusception was diagnosed pre-operatively on repeat CT abdomen and colonoscopy. Given the patient’s large mass with partial obstruction, we decided to proceed with a surgical intervention. The patient underwent an elective robotic-assisted transverse colectomy two weeks after the initial admission and segmental resection of the transverse colon was done with an uneventful post-operative course. Post-operative pathology reports were consistent with submucosal lipoma (5 cm in greatest dimension) with focal fat necrosis and overlying mucosal ulceration without signs of malignancy. Liposarcoma was ruled out after fluorescence in situ hybridization (FISH) analysis for MDM2 gene. 

## Discussion

Intussusception is an uncommon presentation in adults as opposed to children. First described by Paul Barbette, intussusception is an invagination of the proximal portion of the intestine into the distal portion in a telescoping-like fashion, [3]. Intussusception is seen in only 1% to 5% of adult patients [4]. Small bowel intussusceptions are more common than large bowel intussusceptions, accounting for 70% to 80% of cases. Based on the location of intussusception, there are four main types: entero-enteric involving the small intestine, ileocolic involving the ileum and ascending colon, ileocecal in which ileo-ceacal valve act as a lead point, and colo-colonic, involving only the large intestine. Colo-colonic intussusception is rare representing around 17% of all adult intestinal intussusceptions. 

As per the literature review, 60% of the adult-onset intussusception was related to mass-like lesions, only 50% of these masses are malignant. Primary malignant colon cancer accounts for around two-thirds of cases of adult colo-colonic intussusception, the remaining one-third of cases are caused by benign etiologies like adenoma, polyp, lipomas, endometriosis, surgery related, idiopathic [2,5]. The cause of intussusception from lipomas is extremely rare [6]. Lipomas are benign adipose tissue tumors that can occur anywhere in the body; the incidence of colonic lipomas ranges from 0.2% to 4% [7]. Most intestinal lipomas are identified as incidental findings during surgery or colonoscopy, and they are usually asymptomatic. However, large lipomas can be symptomatic. Most common symptoms include abdominal pain, colonic obstruction, bleeding, and intussusception. Due to the slow growth rate of lipomas, it can initially present as abdominal pain which can slowly evolve into partial or complete bowel obstruction, as seen in our patient who has persistent abdomen pain which progressively worsened over a month. Intussusception causing complete bowel obstruction is seen in less than 20% of cases and large lipomas greater than 2-4 cm can cause intestinal obstruction [2,8]. Early diagnosis is difficult in adults as they present with non-specific symptoms. There are multiple imaging modalities for diagnosing colo-colonic intussusception including CT scan, ultrasound, and MRI. CT scan is considered as the imaging modality of choice. It can also reveal benign or malignant masses like lead points. CT scan has a high sensitivity of around 70%-80% and specificity of around 95%-100% to identify intussusception, it can identify three structures including the intestinal wall, mesentrium, and telescoping intestine [9]. CT is also considered the best modality for the diagnosis of colonic lipomas, particularly for lipomas larger than 2 cm. Ultrasound imaging also has a high sensitivity for diagnosing intussusception. The review performed by Honjo et al. demonstrated that combining ultrasound and CT scan had increased the sensitivity to 95.5% [3]. CT and US imaging can show the classic target sign or 'pseudo-kidney' sign. MRIs can also identify benign masses like lipomas and malignant lesions as lead points. Colonoscopy is another modality that can directly visualize intussusception and identify lead points like benign/malignant masses; lipomas usually appear as yellow smooth mass, which can be sessile or angulated [7]. 

Submucosal colonic masses include a variety of differentials like benign lipomas, liposarcoma, leiomyosarcomas, carcinoid, neurofibroma, myoblastoma, etc. [10]. Although endoscopy shows the gross nature of the lesion, it is not diagnostic. The lesions can be malignant (primary or metastases); therefore, it is difficult to exclude without histologic examination of the lesion. In a case report from 2019, pathology from post-operative resection of a submucosal colonic lipoma revealed adenoma with low-grade dysplasia overlying the lipoma. In addition, there were several similar cases with adenomas or pre-malignant lesions [11]. The mechanism behind this is unclear but could be due to pressure-related trauma from stools or recurrent intussusceptions leading to hyperplastic and adenomatous changes [12]. In our patient, a preoperative biopsy was performed to accurately diagnose the lesion and rule out the malignancy. In our clinical scenario, resection is the ideal choice except in children who can be treated with air or saline enemas. Literature review recommended surgical resection of lipomas greater than 2 cm and for lipomas less than 2 cm, endoscopic resection can be done [13]. As large bowel intussusceptions are more likely associated with malignant etiology, surgical resection should be considered as the treatment of choice [14]. Reduction of intussusception via colonoscopy can also be done but it is not recommended as per literature search [15]. Different surgical treatment options include resection of mass lesion, a partial colectomy, hemicolectomy, or subtotal colectomy. Choice of the surgical intervention depends on the size of the lipoma, location of the lipoma, and extent of the intussusception [3]. Our patient did undergo a partial colectomy. 

Prognosis usually varies and depends on a variety of factors like age of the patient, duration for diagnosis, location of intussusception, size of lead point (>2cm needs surgical intervention), and type of mass (benign vs malignant). Pre-operative complications include bowel ischemia, necrosis, perforation, peritonitis, and severe sepsis. These complications are usually life-threatening; delay in diagnosis further worsens the complications, leading to high morbidity and mortality rates [16,17]. Although there are post-operative consequences like superficial wound infection, pneumonia, sepsis, anastomotic leaks, and abscess, these are unavoidable in any intra-abdominal surgery and can be prevented with appropriate measures. 

## Conclusions

In conclusion, although this kind of presentation is rare in general practice, clinicians should be aware of such scenarios and need to decide on an immediate surgical referral. Early diagnosis draws early surgical attention and can be managed based on surgeons' discretion. The benefits of early diagnosis are that we could plan for elective surgery which has fewer post-op complications when compared to emergency laparotomies. Our patient is an ideal example of missed intussusception on initial imaging leading to misinterpretation of patient’s abdominal and had to undergo a hysterectomy. There is still a lot of debate on use of conservative management in intussusception, however, surgical resection remains the mainstay especially in adults. 
